# Differences in Diversity of Collembola Communities Between Primary and Secondary Forests and Driving Factors

**DOI:** 10.3390/insects16080853

**Published:** 2025-08-17

**Authors:** Mingxin Zheng, Zhijing Xie, Yueying Li, Zhuoma Wan, Haozhe Shi, Liping Wang, Qiaoqiao Ji, Zhaojun Wang, Donghui Wu

**Affiliations:** 1 Key Laboratory of Vegetation Ecology, Northeast Normal University, Ministry of Education, Changchun 130024, China; mingxinzheng@nenu.edu.cn (M.Z.); liyy654@nenu.edu.cn (Y.L.); wanzm984@nenu.edu.cn (Z.W.); shihaozhe520@nenu.edu.cn (H.S.); wudonghyi@neiqae.ac.cn (D.W.); 2 State Environmental Protection Key Laboratory of Wetland Ecology and Vegetation Restoration, School of Environment, Northeast Normal University, Changchun 130024, China; 3 State Key Laboratory of Black Soils Conservation and Utilization, Northeast Institute of Geography and Agroecology, Chinese Academy of Sciences, Changchun 130102, China; wangliping221@mails.ucas.ac.cn; 4 Chengdu Natural History Museum, Chengdu University of Technology, Chengdu 610059, China; qiaoqiao_ji@163.com

**Keywords:** forest conversion, soil fauna, springtails, biodiversity conservation, community structure

## Abstract

Primary forests harbor extraordinary biodiversity, but conversion from primary forests to secondary forests often leads to biodiversity loss and diminished ecosystem functioning. While much of the existing research has focused on plants and vertebrates, soil fauna—particularly Collembola—remain underexplored in this context. To address this gap, we conducted a comprehensive assessment of the Collembola diversity and community composition in primary and secondary forests across two regions in northeastern China. Among 5587 Collembola individuals, 69 morphospecies were identified. The Collembola abundance and Shannon–Wiener index were significantly higher in primary forests, although the species richness did not differ significantly between the forest types. In contrast, the community composition differed markedly, with several taxa found exclusively in primary forests. Notably, environmental factors exerted stronger influences on Collembola communities in primary forests, suggesting that these ecosystems may be more vulnerable to climate change and external disturbances. These findings demonstrate that primary forests play a crucial role in protecting soil fauna diversity and emphasize that future conservation efforts should focus on the strict protection of primary forests.

## Simple Summary

The conversion of primary forests to secondary forests often leads to biodiversity loss and diminished ecosystem functioning. However, the impact of this transformation on soil fauna—particularly Collembola, one of the most widespread and ecologically important arthropods—remains poorly understood. To address this gap, we conducted a comprehensive assessment of the Collembola diversity and community composition in primary and secondary forests across two regions in northeastern China. Our results showed that primary forests supported a significantly higher Collembola abundance and Shannon–Wiener index than secondary forests. Moreover, some rare Collembola groups were only found in primary forests, highlighting their importance for conserving unique soil biodiversity. Notably, Collembola communities in primary forests were more sensitive to environmental factors such as the soil nutrients and climate, indicating that changes in the climate or habitat quality may have a stronger impact on these communities in primary forests. These findings highlight that protecting primary forests is crucial for maintaining soil health and biodiversity. At the same time, secondary forests also support many soil species and should also be included in conservation efforts.

## 1. Introduction

Primary forests are essential for biodiversity conservation and play an important role in carbon sequestration and climate change mitigation [1,2,3]. However, these ecosystems are increasingly threatened by deforestation and land use change, with less than 25% of the global primary forest area remaining undisturbed [4,5,6]. As primary forests decline, secondary forests are becoming increasingly dominant worldwide. While the biodiversity of primary forests has been extensively documented—especially for plants and birds, which are often more diverse in primary than in secondary forests [7,8]—belowground biota remain comparatively understudied, despite their fundamental ecological roles.

Soil fauna, as an indispensable component of forest ecosystems, contribute significantly to litter decomposition, nutrient cycling, and the regulation of microbial communities [9,10,11]. These organisms are highly responsive to forest succession and are generally more diverse in primary forests, likely due to more favorable and stable microclimatic conditions [12,13,14]. For example, the richness and abundance of soil fauna in tropical mountain Andean forests increase with succession [12]. Similarly, research in the Amazon rainforest indicates that dung beetles are more abundant in primary than in secondary forests [15].

In addition to the species diversity, the soil fauna community composition also varies significantly between primary and secondary forests [16,17]. These compositional differences are likely driven by shifts in microhabitat complexity associated with the forest structure and environmental factors. The more structurally diverse vegetation in primary forests supports a wider array of microhabitats than do the more uniform canopies of secondary forests [18,19,20,21]. For instance, arboreal ant communities in secondary forests diverge significantly from those in primary forests due to their simpler vegetation structure and lower turnover of nesting microhabitats between the trees [22]. Additionally, soil fauna are strongly influenced by environmental factors such as the soil moisture, temperature, and pH [23,24]. In tropical and temperate forests alike, the soil pH and moisture have been shown to shape macrofaunal assemblages during forest transformation [25]. Notably, the effects of these environmental variables may be more pronounced in secondary forests due to their early successional stage, characterized by sparse understory vegetation, thinner litter layers, and less stable microhabitats. Such conditions reduce the buffering capacity of these forests against environmental fluctuations, making soil fauna more vulnerable to changes [1,26,27,28].

Collembola are among the most widespread arthropods, inhabiting nearly all terrestrial ecosystems, and play vital roles in ecosystem processes such as carbon and nitrogen cycling, soil microstructure formation, and plant litter decomposition [29,30,31]. Due to their rapid responses to environmental changes, Collembola are widely recognized as effective bioindicators of forest disturbances and succession [32,33,34]. In temperate regions, studies have shown that both the diversity and community composition of Collembola differ between primary and secondary forests, with higher diversity typically found in the former [35,36]. These patterns are influenced by environmental factors such as the soil pH [37], nutrient availability (e.g., of carbon and phosphorus [38]), and climatic variables like the temperature and precipitation [39,40]. Moreover, Collembola communities in primary forests may be less affected by environmental variability due to the ecosystem’s greater capacity to buffer against fluctuations and maintain soil stability [21,41,42]. Therefore, considering the environmental context is crucial to understanding the mechanisms that shape the Collembola diversity and distribution, with implications for forest conservation and restoration.

In this study, we compared Collembola communities in primary and secondary forests within northeastern China’s temperate mixed Korean pine–broadleaf forests. Specifically, we investigated differences in the species diversity, community composition, and environmental drivers shaping these communities. We addressed the following hypotheses:

**H1** : *The Collembola diversity (richness, abundance, and Shannon–Wiener index) is higher in primary forests than in secondary forests*.

**H2** : *The community composition differs significantly between primary and secondary forests.*

**H3** : *Collembola communities in secondary forests exhibit stronger responses to environmental variables than those in primary forests*.

## 2. Materials and Methods

### 2.1. Study Area

This study was conducted in the Huangnihe Nature Reserve (127°54′~128°14′ E, 43°55′~44°06′ N) in the Yanbian Korean Autonomous Prefecture, Jilin Province, and the Fangzheng Twin Mountain Primeval Forest Park (129°6′ E, 45°3′ N) in Harbin, Heilongjiang Province, both located in northeast China. The Huangnihe Nature Reserve and Fangzheng Twin Mountain Primeval Forest Park cover areas of 41,583 and 680 ha, respectively [43,44]. Both sites exhibit high forest coverage exceeding 88% and are characterized by a typical temperate monsoon climate. The mean annual temperatures are approximately 2.6 °C, and the mean annual precipitations are around 600 mm. The summers are warm and humid, while the winters are cold and dry. The primary forests at both sites are well-preserved, mature mixed Korean pine–broadleaf forests, dominated by *Pinus koraiensis* and aged over 100 years. In contrast, the secondary forests have naturally regenerated over more than 30 years following historical logging and are dominated by deciduous species such as *Betula platyphylla*, *Populus davidiana*, *Quercus mongolica*, and *Phellodendron amurense* (Table S1). Although their species composition differs, these patterns reflect typical successional trajectories in northeastern China, allowing us to evaluate the biodiversity’s responses to realistic forest conversion processes. The sampling plots in Fangzheng and Huangnihe were located at altitudes of approximately 428 m and 609 m, respectively. The terrain was characterized by a relatively high elevation and steep slopes. The geological substrate in both regions is dominated by granite formations, which have weathered into dark brown forest soils rich in organic matter, typical of the temperate forest zone in northeastern China [44,45] (Table S1).

### 2.2. Sampling Procedure

Sampling was conducted in early September, during the late growing season, when the Collembola activity and diversity typically peak in this region, ensuring the collection of representative data on their active communities. All the sampling plots were situated within national nature reserves, where the forest ecosystems were protected from industrial and agricultural activities. The sites were not subject to ongoing human disturbances and were managed solely for conservation and scientific purposes. Litter and soil samples were collected from five randomly selected 100 m^2^ plots in primary and secondary forests at Fangzheng and Huangnihe. The five sample plots were spaced at least 100 m apart to ensure spatial independence. In each plot, three subplots were randomly selected for litter and soil sampling and subsequently pooled to form one replicate. Litter samples were collected using a 100 cm^2^ frame, and soil samples were extracted from directly beneath the litter layer using a soil core (5.5 cm diameter, to a depth of 10 cm). In total, 40 samples were obtained (2 locations × 2 forest types × 2 layers × 5 replicates). All the samples were transported to the laboratory within 24 h and processed using Berlese–Tullgren funnels over a 10-day extraction period. The extracted Collembola were preserved in 95% ethanol for further identification and analysis.

### 2.3. Morphospecies Identification

The Collembola individuals were sorted and their morphospecies identified based on external morphological features using a stereomicroscope and the available taxonomic literature. Here, ‘morphospecies’ refer to species-level units recognized sensu stricto, using classical morphological taxonomy. Due to a limited regional taxonomic resolution, many morphospecies could not be assigned valid species names but were treated as distinct taxa in all the analyses. For each morphospecies identified within a sample, at least eight individuals were selected, cleaned with lactic acid, mounted in Hoyer’s solution, and examined under a Zeiss Axio Scope A1 compound microscope for detailed morphological analysis. Collembola were identified at the morphospecies level based on taxonomic references, including the *Pictorial Keys to Soil Animals of China* [46], *Synopses on Palaearctic Collembola* [47], and the online database https://www.collembola.org/ (accessed on 31 October 2024) [48]. Whenever possible, immature specimens were sorted into morphospecies by comparing them with the corresponding adults or subadults.

### 2.4. Environmental Variables

To identify the potential drivers of Collembola communities in both primary and secondary forests, we selected two categories of environmental variables: local habitat-related factors and climatic variables. The local habitat-related factors were measured in the soil and litter samples after extraction of the soil fauna. The samples were homogenized using a ball mill and analyzed for five variables: the total carbon (TC), total nitrogen (TN), total phosphorus (TP), pH, and carbon-to-nitrogen (C/N) ratio. The soil and litter pH were analyzed using a pH meter (PHS-3C, LEICI, China) with an aqueous suspension (soil: water = 1:2.5 wt/vol; litter: water = 1:8 wt/vol) [49]. The TC and TN were determined using an elemental analyzer (vario MACRO cube, Elementar, Germany), while the TP was measured through H_2_SO_4_-HClO_4_ digestion. The C/N ratio was calculated from the measured TC and TN values for both the litter and soil. The climatic variables included the mean annual temperature (MAT) and mean annual precipitation (MAP), which were downloaded from the *WorldClim* global database (1 km^2^ resolution, https://worldclim.org).

### 2.5. Statistical Analysis

The species abundance in the litter and soil at each sampling point was standardized to a 1 m^2^ area. All statistical analyses were performed using R version 4.3.3 (R Core Team, 2024), with visualizations created using the “ggplot2” package. Species accumulation curves were generated with the “specaccum” function from the “vegan” package, and sample completeness curves for the primary and secondary forests were plotted using the “iNEXT” package to evaluate the sampling sufficiency [50] (Figure S1).

To assess differences in the environmental factors and Collembola diversity between the primary and secondary forests, a one-way analysis of variance (ANOVA) was conducted. The data normality and homogeneity of variances were tested using the Shapiro–Wilk Test and Levene’s Test, respectively, using the “car” package. The Collembola diversity was estimated using the abundance, richness, and Shannon–Wiener Index. The Shannon–Wiener index was selected because it is influenced by both the richness and evenness and is more sensitive to changes in the abundance of rare groups. As the diversity indices did not differ significantly between Fangzheng and Huangnihe (Figure S2), the geographical location was excluded from the subsequent analysis.

To compare the Collembola community composition between the primary and secondary forests, we performed permutational multivariate analyses of variance (PERMANOVAs) and principal coordinate analysis (PCoA) using Bray–Curtis dissimilarity matrices, employing the “vegdist” and “adonis” functions in the “vegan” package [51]. Notably, PCoA was conducted not only on the total communities but also separately on the Collembola communities in the litter and soil layers across the different locations. The species overlap between the primary and secondary forests was visualized with Venn diagrams using the “VennDiagram” package. Finally, differences in the relative abundance of Collembola families were illustrated using the “ggalluvial” package.

To evaluate the environmental drivers of Collembola diversity, Pearson correlation analyses were conducted between the diversity indices (Shannon–Wiener index, richness, abundance) and environmental variables using the “rcoor” function in the “Hmisc” package. Then, to further investigate the influence of the environmental variables on the Collembola community in primary and secondary forests, we first assessed the correlations among the environmental variables by performing Pearson correlation analyses. Variables with correlation coefficients greater than 0.80 were considered strongly correlated and were removed from further analysis [52]. As a result, eight factors were retained for the primary forests (soil and litter pH and C/N ratios, soil TN, soil TP, litter TC, and mean annual precipitation) and seven factors for the secondary forests (same as above, excluding the litter TC). Redundancy analysis (RDA) was then performed to examine the relationship between the Collembola community composition and environmental factors. Before performing the RDA, community data were standardized using “Hellinger” transformation and environmental data using “standardize” from the function “decostand” in the “vegan” package. RDA was performed on non-collinear factors, following the forward selection model with the “ordistep” function from the “vegan” package. The overall significance of the models was evaluated using Monte Carlo tests with 999 permutations. The proportion of the variation explained by the selected environmental variables was quantified using adjusted R^2^ values. Notably, RDAs were conducted separately on the relationships between the environmental factors and Collembola community composition in both the primary and secondary forests. Then, to evaluate the unique contribution of each environmental factor we applied the “rdacca.hp” function from the “rdacca.hp” package [53].

## 3. Results

A total of 5587 Collembola individuals were collected and identified, representing 69 species or morphospecies across nine families. Among these, 56 species and nine families were recorded in the primary forest, while 40 species and seven families were found in the secondary forest. The species accumulation curves became marginally asymptotic, indicating that most species present at both Fangzheng and Huangnihe had been sampled (Figure S1a). Additionally, the rarefaction curves reached asymptotic plateaus, suggesting that the sampling effort sufficiently captured the species diversity of the Collembola communities at both study sites (Figure S1b). Although our sampling was conducted during the peak of Collembola activity in early September, the one-time design does not account for temporal variation. Future research should incorporate repeated or seasonal sampling to better capture the temporal dynamics and enhance the generalizability of biodiversity patterns across forest types.

### 3.1. Litter and Soil Properties and Collembola Species Diversity

The litter TN and TP in primary forests were significantly lower than those in secondary forests in Fangzheng and Huangnihe, and the litter C/N ratio was significantly higher in primary forests compared to secondary forests in Fangzheng, but not in Huangnihe (Table 1). Additionally, no significant differences were found in the other soil properties.

**Figure 1:**
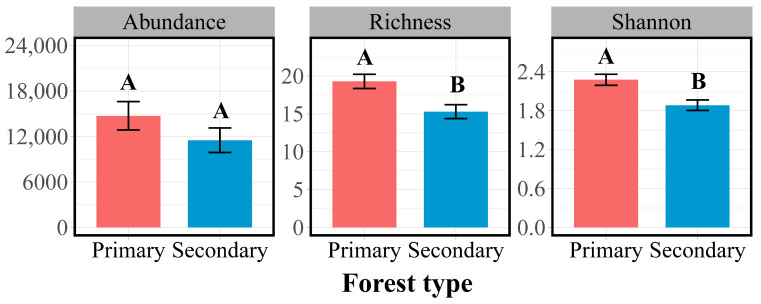
Abundance (ind. m^−2^), richness, and Shannon–Wiener index of Collembola communities in primary and secondary forests. Different letters indicate significant differences between forest types based on ANOVA test (*p* < 0.05).

The Collembola abundance and Shannon–Wiener index were significantly higher in primary forests than in secondary forests (Figure 1), with these differences mainly driven by the soil layer. In both forest types, the species richness, abundance, and Shannon–Wiener index were significantly higher in the litter layer than in the soil layer (Table S2). However, no significant difference in the Collembola abundance was observed between the litter and soil layers within either forest type (Figure S2).

### 3.2. Community Composition of Collembola

**Figure 2:**
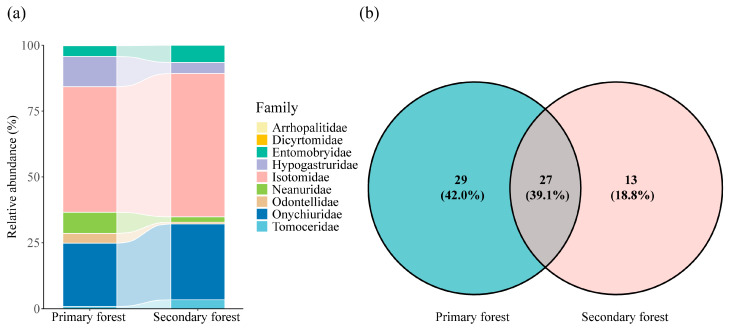
Relative abundance of Collembola families in primary and secondary forests (**a**) and the overlap in the species between the two forest types (**b**).

The primary and secondary forests shared 27 species (39.1% of all the recorded species), with 29 species (42.0%) found exclusively in the primary forests and 13 species (18.8%) found only in the secondary forests (Figure 2b). Several morphospecies showed clear habitat preferences linked to the forest type. *Arrhopalites* sp. 12 (Arrhopalitidae) and *Dicyrtoma* sp. 2 and sp. 3 (Dicyrtomidae) were exclusively found in primary forests, suggesting their association with undisturbed habitats (Table S4). In contrast, thirteen morphospecies occurred only in secondary forests, including members of Entomobryidae, Neanuridae, and Isotomidae (Figure 2b). These patterns indicate that the forest use history significantly influences the Collembola community composition through a taxon-specific response. At the family level, primary forests exhibited greater family richness (nine families) compared to secondary forests (seven families), with Arrhopalitidae and Dicyrtomidae being unique to primary forests (Figure 2a). Isotomidae predominated in both forest types, with its relative abundance being approximately 7% higher in secondary forests (54.5%) compared to primary forests (47.7%). The relative abundances of Entomobryidae, Onychiuridae, Isotomidae, and Tomoceridae were higher in secondary forests, whereas those of Hypogastruridae, Neanuridae, and Odontellidae were higher in primary forests.

**Figure 3:**
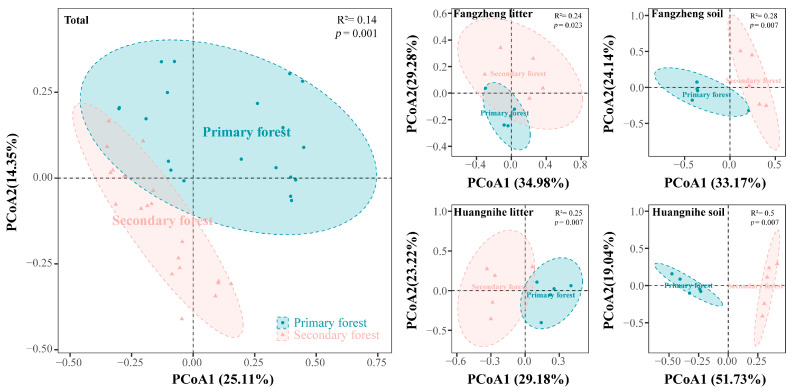
Community composition of Collembola in primary and secondary forests as indicated by a principal coordinate analysis (PCoA). The analysis was conducted separately based on the total data and those for the litter and soil layers from different locations.

Significant differences in the community composition were observed between primary and secondary forests across all the samples (Figure 3, Table S3). The first two axes of the PCoA explained 39.46% of the total variation in the community composition. Significant differences in the community composition were also observed between primary and secondary forests within both the litter and soil layers at the two locations.

### 3.3. The Drivers of the Collembola Diversity and Species Composition

**Figure 4:**
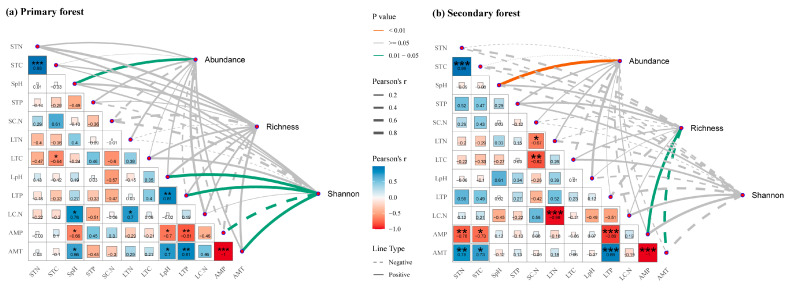
Pearson correlation coefficients between environmental factors studied, as well as between environmental factors and species diversity of Collembola in primary (**a**) and secondary forests (**b**). STN: soil total nitrogen; STC: soil total carbon; SpH: soil pH; STP: soil total phosphorus; SC.N: soil C/N ratio; LTN: litter total nitrogen; LTC: litter total carbon; LpH: litter pH; LTP: litter total phosphorus; LC.N: litter C/N ratio; AMT: mean annual temperature; AMP: mean annual precipitation. * 0.01 < *p* < 0.05, ** 0.001 < *p* < 0.01, *** *p* < 0.001.

The Collembola abundance was positively correlated with the soil pH in both primary and secondary forests (Figure 4). In secondary forests, the species richness showed significant positive correlations with the MAP and a negative relationship with the MAT, while no significant correlations were found in primary forests. In contrast, the Shannon–Wiener index in primary forests was positively associated with the litter pH, litter TP, and MAT and negatively associated with the MAP, while no significant relationships were observed in secondary forests.

**Figure 5:**
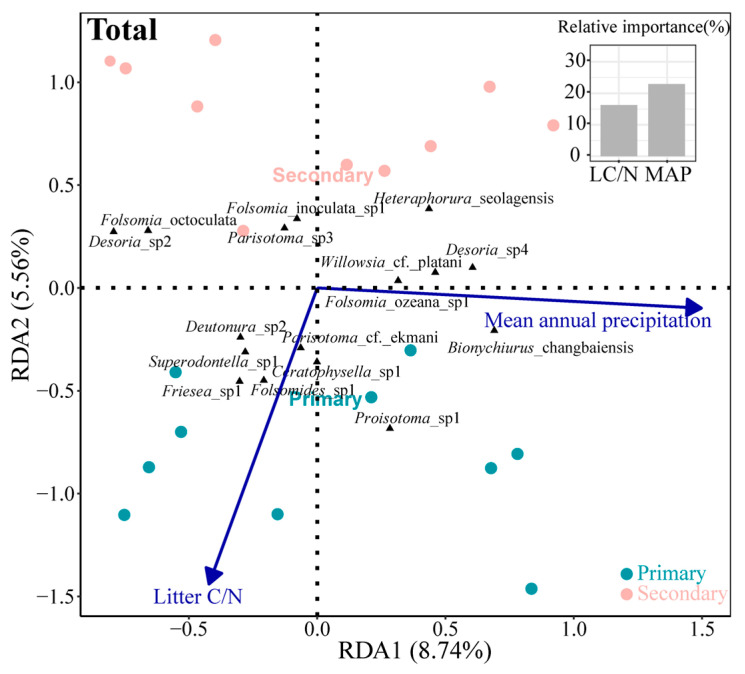
Redundancy analysis of the relationship between the Collembola species and environmental factors across all the samples. The length of the arrows represents the percentage of the variation explained by each respective variable. Triangles represent species scores in the RDA ordination space.

Across both forest types, the litter C/N ratio and MAP were the main environmental variables driving the community composition (Monte Carlo test, *p* < 0.05). The first two axes of the RDA explained 14.30% of the total variation in the Collembola community composition, with the first axis accounting for 8.74% and the second axis for 5.56%. Among the selected variables, the MAP explained a greater proportion of the variation than the litter C/N ratio (23.39% vs. 15.63%; Figure 5).

**Figure 6:**
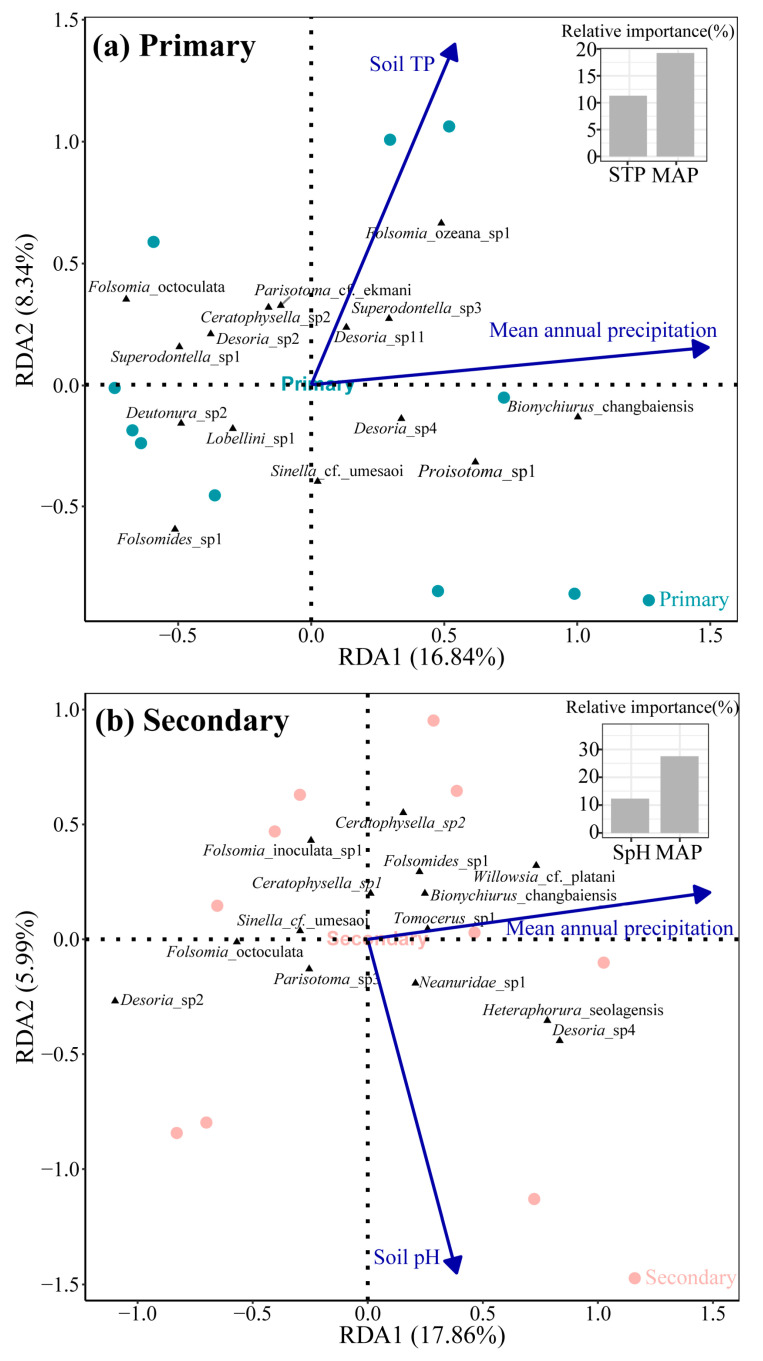
Redundancy analysis of the relationship between the Collembola species and environmental factors across primary (**a**) and secondary forests (**b**). The length of the arrows represents the percentage of the variation explained by each respective variable. Triangles represent species scores in the RDA ordination space.

The drivers of the Collembola community composition differed between primary and secondary forests. In the primary forest, the soil TP and MAP were the key drivers (Monte Carlo test, *p* < 0.05), and the first two RDA axes explained 25.17% of the variation. Species such as *Folsomia ozeana* sp.1, *Desoria* sp.11, and *Superodontella* sp.3 were more prevalent in environments with a higher soil TP and MAP. Notably, the MAP explained more variation than the soil TP (19.23% vs. 12.59%; Figure 6a). In secondary forests, the soil pH and MAP were the primary drivers (Monte Carlo test, *p* < 0.05), with the first two RDA axes explaining 23.85% of the variation. *Tomocerus* sp.1 and *Bionychiurus changbaiensis* were associated with areas with a higher MAP, while *Folsomia inoculate* sp.1 and *Ceratophysella* sp.1 were more common in soils with a lower pH. Again, the MAP accounted for a larger share of the explained variation than the soil pH (26.12% vs. 12.05%; Figure 6b).

## 4. Discussion

### 4.1. Differences in Collembolan Diversity Between Primary and Secondary Forests

Partially confirming the first hypothesis, the Collembola abundance and Shannon–Wiener index were higher in primary forests compared to secondary forests, while the species richness did not differ significantly between the two forest types. This pattern aligns with findings from the Changbai Mountains, where primary mixed coniferous–broadleaf forests supported a greater Collembola abundance and diversity compared to secondary forests [54]. Similarly, Boonrotpong et al. (2004) reported a significantly higher abundance and Shannon–Wiener diversity of dung beetles in primary compared to secondary forests in the tropical Ton Nga Chang Wildlife Sanctuary, Thailand [55]. These patterns suggest that undisturbed primary forests provide more favorable environmental conditions and resource availability for soil fauna.

In contrast, the species richness did not differ significantly between primary and secondary forests. This finding aligns with previous research in both temperate and tropical ecosystems. For example, Addison et al. (2003) reported minimal variation in the arthropod richness across successional stages of Vancouver Island forests [36]. Comparable richness patterns have also been found in ant communities in Andean tropical forests, where primary and secondary stands—particularly at lower elevations—supported similar levels of species richness [56]. Likewise, during subalpine secondary succession, Zhang et al. (2023) found no significant difference in the soil mite richness between primary and secondary forests [57]. These findings suggest that the species richness may not be as sensitive to forest disturbances as other diversity indices like the abundance or Shannon–Wiener index.

### 4.2. Differences in Collembolan Community Composition Between Primary and Secondary Forests

In support of the second hypothesis, the Collembola community composition differed significantly between primary and secondary forests. Similar patterns of results have been obtained in other soil fauna. For instance, Bitencourt et al. (2019) reported differences in the community composition of dung beetles between primary and early successional forests in the Amazon tropical rainforest [15]. Similarly, Wang et al. (2023, 2025) demonstrated that nematode and microbial communities (bacteria and fungi) shift notably between early and late successional forests, largely driven by changes in the dominant plant species and associated environmental conditions [58,59].

At the family level, the community composition also differed markedly between primary and secondary forests. Two families—Arrhopalitidae and Dicyrtomidae—were found exclusively in primary forests; these families are generally associated with more stable, less disturbed environments and likely represent habitat specialists or rare taxa (Arrhopalitidae and Dicyrtomidae accounted for 0.10% and 0.07% of all the individuals collected, respectively) [60,61,62]. Their absence in secondary forests may reflect habitat simplification and reduced environmental suitability following disturbances. Our findings suggest that the forest disturbance history plays a strong filtering role in shaping Collembola communities. The exclusive occurrence of some morphospecies (e.g., *Arrhopalites* sp. 12, *Dicyrtoma* spp.) in primary forests highlights their sensitivity to anthropogenic disturbances and potential dependence on long-term habitat stability. Conversely, the presence of taxa unique to secondary forests may reflect tolerance or adaptation to altered environmental conditions. These results align with previous research emphasizing the impact of the habitat legacy on soil fauna assemblages. While our identifications were primarily at the morphospecies level, the distinct distribution patterns observed suggest meaningful ecological differentiation. Future studies incorporating molecular taxonomy and long-term monitoring could provide deeper insights into species-specific responses to the forest management and disturbance history. By contrast, Isotomidae was the dominant family in both primary and secondary forests; a similar result was also found in beech forest succession research [63]. The dominance of Isotomidae is likely attributable to its broad ecological tolerance and strong dispersal ability [64,65,66]. These findings emphasize the importance of preserving primary forests to protect specialized or less disturbance-tolerant soil arthropod species.

### 4.3. Relationship with Environmental Variables in Primary and Secondary Forests

Contrary to the third hypothesis, the Collembola community in primary forests exhibited a stronger response to the environmental variables than those in secondary forests. Specifically, the Shannon–Wiener index was more responsive to environmental factors in primary forests compared to secondary forests. This was likely due to the higher species richness and evenness of Collembola observed in these forests (Figure 2), which indicate a more complex community structure where changes in the environmental conditions can cause shifts in dominance and interspecific dynamics [67]. A decline in environmental quality may lead to the dominance of a few tolerant species, thereby reducing the overall diversity. Notably, the mean annual temperature (MAT) and precipitation (MAP) significantly influenced the Collembola species richness in secondary forests but had limited effects in primary forests. This suggests that secondary forests, which are often characterized by reduced vegetation structure complexity and microclimatic buffering, are more sensitive to climatic fluctuations [21,42,68]. As a result, Collembola in these habitats may be more vulnerable to microclimatic fluctuations, even though their overall diversity metrics are lower.

The Collembola community composition also responded more strongly to environmental variables in the primary forest, where the soil total phosphorus (TP) emerged as a key driver. This may have been due to the role of the TP in supporting microbial communities, an essential food source for Collembola. The soil microbial composition and activity are closely linked to the phosphorus availability [69], and the TP typically declines during forest succession [70,71], making it a limiting resource [72]. Consequently, in primary forests, the soil TP likely influences Collembola indirectly through its effects on the microbial food availability. By contrast, the soil pH emerged as the dominant factor influencing the Collembola composition in secondary forests. The soil pH often co-varies with other key properties such as the organic matter, base cation concentration, and nitrification potential [73,74], all of which can affect the Collembola distribution and abundance [75]. Thus, the pH may act as a proxy for broader soil quality conditions in these more disturbed environments.

In both primary and secondary forests, climatic factors such as precipitation also played an important role in shaping Collembola communities. The climate influences soil fauna communities through both direct physiological constraints and indirect pathways, including the vegetation structure and trophic interactions [76]. In this study, widely distributed Collembola families such as Isotomidae (e.g., *Folsomia ozeana* sp.1 and *Desoria* sp.11) and Onychiuridae (*Bionychiurus changbaiensis*) were particularly associated with high precipitation. Precipitation interacts with the soil structure and microbial communities, which can subsequently affect the composition of microarthropods, including Collembola [77].

## 5. Conclusions

Our results indicate that the Collembola abundance and Shannon–Wiener index are significantly higher in primary forests than in secondary forests, and that the community composition differs markedly between the two forest types. Notably, two families—Arrhopalitidae and Dicyrtomidae—were found exclusively in primary forests, underscoring the importance of undisturbed habitats in conserving specialized and potentially vulnerable soil fauna. Moreover, Collembola communities in primary forests exhibited stronger associations with environmental factors, indicating their heightened sensitivity to ecological changes. These results demonstrate that primary forests serve as irreplaceable refuges for specialized and endemic soil fauna, the loss of which could compromise key ecosystem processes. The heightened environmental sensitivity of these communities underscores the urgent need to prioritize primary forest conservation. At the same time, secondary forests still maintain considerable soil fauna diversity and should not be neglected in conservation strategies. This dual approach—prioritizing primary forest protection while recognizing the value of secondary forests—offers the most comprehensive framework for preserving soil biodiversity across forest landscapes.

## Figures and Tables

**Table 1:** Mean (± standard error, SE) of the environmental variables measured in the litter and soil layers of primary and secondary forests in Fangzheng and Huangnihe.

Environmental Factor	Site	Depth	Forest		Summary of ANOVA Results	
			Primary	Secondary	F Test	*p* Value
Total N (%)	Fangzheng	Litter	1.07 ± 0.29 **^a^**	1.66 ± 0.27 **^b^**	10.802	**0.011**
		Soil	0.61 ± 0.18	0.92 ± 0.4	2.437	0.157
	Huangnihe	Litter	1.2 ± 0.13 **^a^**	1.57 ± 0.26 **^b^**	7.797	**0.023**
		Soil	0.6 ± 0.23	0.38 ± 0.05	3.973	0.081
Total C (%)	Fangzheng	Litter	44.77 ± 1.44	44.77 ± 1.68	0.200	0.667
		Soil	7.32 ± 2.49	12.11 ± 6.55	2.290	0.169
	Huangnihe	Litter	41.83 ± 8.95	44.65 ± 0.46	0.848	0.384
		Soil	7.9 ± 3.53	5.16 ± 0.94	2.138	0.182
Total P (g/kg)	Fangzheng	Litter	4.39 ± 0.63 **^a^**	5.49 ± 0.59 **^b^**	8.030	**0.022**
		Soil	1.42 ± 0.27	1.77 ± 0.98	0.581	0.468
	Huangnihe	Litter	3.12 ± 0.37 **^a^**	3.75 ± 0.36 **^b^**	7.396	**0.026**
		Soil	1.8 ± 0.51	1.59 ± 0.3	0.575	0.470
pH	Fangzheng	Litter	5.72 ± 0.13	5.88 ± 0.19	1.980	0.197
		Soil	5.52 ± 0.31	5.2 ± 0.19	3.879	0.084
	Huangnihe	Litter	5.5 ± 0.12	5.96 ± 0.82	0.001	0.972
		Soil	5.07 ± 0.26	5.26 ± 0.36	0.979	0.352
C/N ratio	Fangzheng	Litter	45.3 ± 16.17 **^a^**	27.51 ± 4.18 **^b^**	8.219	**0.021**
		Soil	11.88 ± 0.92	12.85 ± 1.58	1.419	0.268
	Huangnihe	Litter	34.48 ± 5.36	29.02 ± 4.52	2.792	0.133
		Soil	12.87 ± 2.35	13.02 ± 0.62	0.019	0.894

Different letters indicate significant differences (*p* < 0.05 based on ANOVA test) between means of primary and secondary forests. Bold values indicate significant statistical differences.

## Data Availability

The data are available on request from the authors.

## Supplementary Materials

The following supporting information can be downloaded at: https://www.mdpi.com/article/10.3390/insects16080853/s1, Figure S1: Species accumulation curves (a) and sample completeness curves with extrapolations (b) for Collembola in primary and secondary forests at Fangzheng and Huangnihe sites. Figure S2: The abundance (ind. m^−2^), richness, and Shannon–Wiener index of Collembola communities in the soil and litter layers of primary and secondary forests. Different letters indicate significant differences between primary and secondary forests based on an ANOVA test, *p* < 0.05. Table S1: Basic information of the study area. Table S2: Analysis of variance (ANOVA) of Collembola diversity (abundance, richness, and Shannon–Wiener index) in relation to different forest types, sites studied, and layers. Table S3: PERMANOVA analysis of the Collembola community composition in primary and secondary forests. Table S4: Collembola species recorded in primary and secondary forests.
