# Supplementary material for: Differences in Diversity of Collembola Communities Between Primary and Secondary Forests and Driving Factors
Source: Insects. 2025 Aug 17;16(8):853. doi: 10.3390/insects16080853 (PMC12386487; doi:10.3390/insects16080853)
Supplement: Supplementary file 1 [file insects-16-00853-s001.zip › insects-3773741-supplementary.pdf]

Supplementary Information

Article

Differences in Diversity of Collembola Communities Between Primary and Secondary Forests and Driving Factors

Mingxin Zheng [1,2], Zhijing Xie [1,2,*], Yueying Li [1,2], Zhuoma Wan [1,2], Haozhe Shi [1,2], Liping Wang [3], Qiaoqiao Ji [4], Zhaojun Wang [1,2,*] and Donghui Wu [1,2,3]

1 Key Laboratory of Vegetation Ecology, Northeast Normal University, Ministry of Education, Changchun 130024, China; mingxinzheng@nenu.edu.cn (M.Z.); liyy654@nenu.edu.cn (Y.L.); wanzm984@nenu.edu.cn (Z.W.); shihaozhe520@nenu.edu.cn (H.S.); wudonghyi@neiqae.ac.cn (D.W.)
2 State Environmental Protection Key Laboratory of Wetland Ecology and Vegetation Restoration, School of Environment, Northeast Normal University, Changchun 130024, China
3 State Key Laboratory of Black Soils Conservation and Utilization, Northeast Institute of Geography and Agroecology, Chinese Academy of Sciences, Changchun 130102, China; wangliping221@mails.ucas.ac.cn
4 Chengdu Natural History Museum, Chengdu University of Technology, Chengdu 610059, China; qiaoqiao_ji@163.com
* Correspondence: xiezhijing@nenu.edu.cn (Z.X.); wangzj217@nenu.edu.cn (Z.W.)

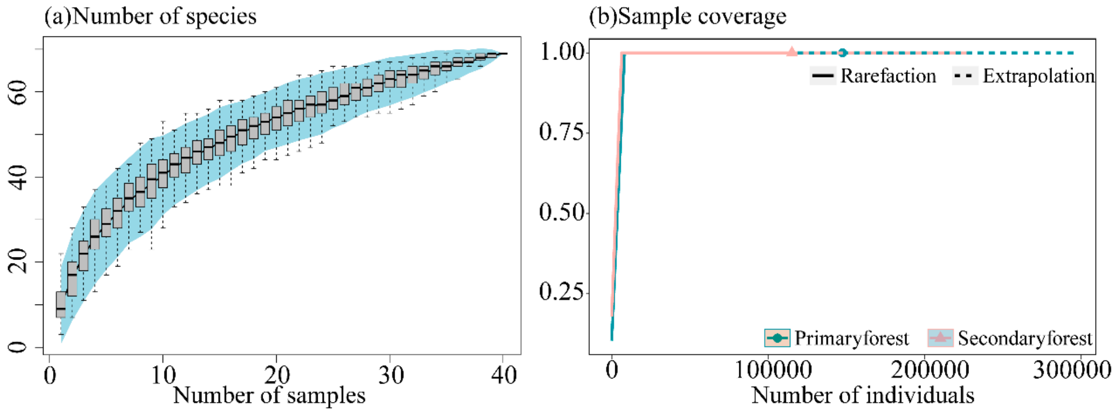

Figure S1. Species accumulation curves (a) and sample completeness curves with extrapolations (b) for Collembola in the primary and secondary forests at Fangzheng and Huangnihe sites.

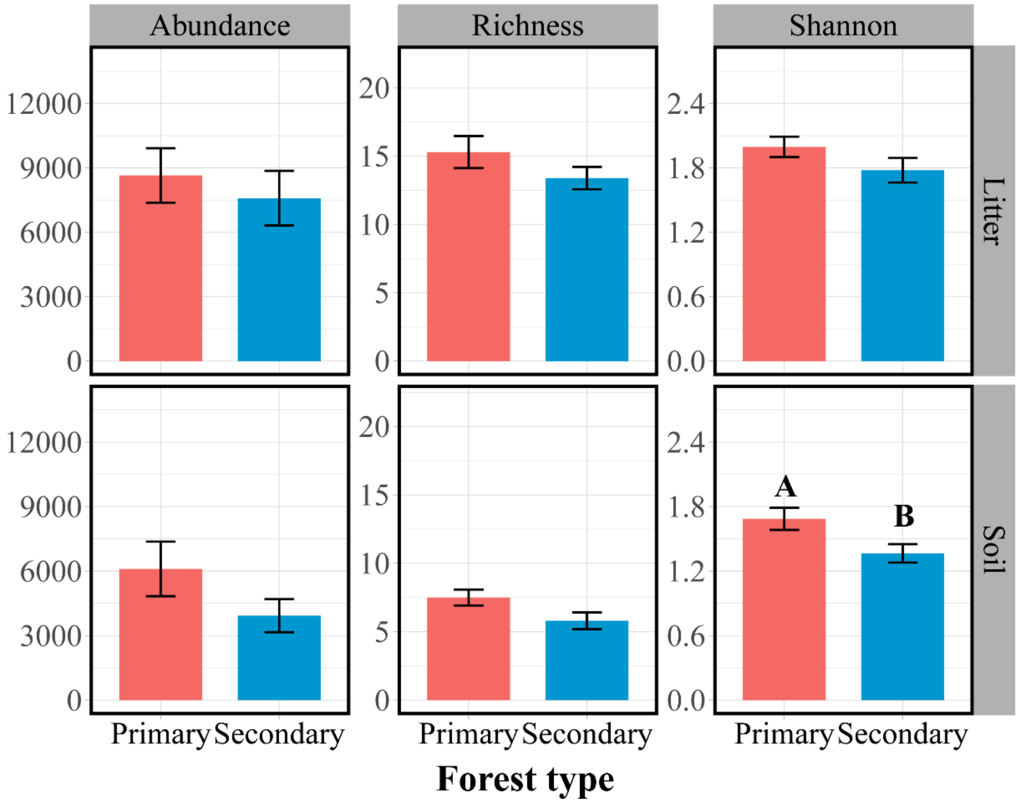

Figure S2. The abundance (ind. m⁻²), richness, and Shannon-Wiener index of Collembola community in the soil and litter layers of primary and secondary forests. Different letters indicate significant differences between primary and secondary forests based on ANOVA test, $P < 0.05$.

Table S1. Basic information of the study area.

Basic information of the study area		
Indices	Huangnihe Nature Reserve	Fangzheng Twin Mountain Primeval Forest Park
Latitude	44°01′48″N	45°39′30″N
Longitude	127°55′41″E	129°5′6″E
Elevation	609	428
Annual mean temperature	2.4℃	2.6℃
Annual mean precipitation	632 mm	596.2 mm
Soil type	Dark brown forest soil	Dark brown forest soil
Dominate tree species in primary forests	*Pinus koraiensis* Siebold & Zucc	*Pinus koraiensis* Siebold & Zucc
Dominate tree species in secondary forests	*Betula platyphylla* Sukaczev, *Populus davidiana* Dode	*Quercus mongolica* Fisch. ex Ledeb, *Phellodendron amurense* Rupr
References	Kun, 2004	Wang, 2018

Table S2 Analysis of variance (ANOVA) of Collembola diversity (abundance, richness and Shannon-Wiener index) in relation to different forest types, studied sites and layers. Signifcant efects indicated in bold.

	Abundance		Richness		Shannon-Wiener	
	F-value	p-value	F-value	p-value	F-value	p-value
(Intercept)	8123.453	<.0001	694.488	<.0001	1303.957	<.0001
Location	0.010	0.921	0.772	0.386	1.182	0.285
Forest	2.559	0.120	5.102	**0.031**	8.119	**0.008**
Layer	8.072	**0.008**	93.370	**<.0001**	14.582	**0.001**
Location : Forest	0.580	0.452	3.543	0.069	6.305	**0.017**
Location : Layer	3.680	0.064	0.567	0.457	0.125	0.726
Forest : Layer	0.405	0.529	0.016	0.901	0.301	0.587
Location : Forest : Layer	0.274	0.604	2.268	0.142	0.750	0.393

Table S3 PERMANOVA analysis of the Collembola community composition in primary and secondary forests.

Group	pairs	Df	SumsOfSqs	F.Model	R^2	p.value	p.adjusted
Total	Primary vs Secondary	1	1.483	6.070	0.138	0.001	**0.001**
Fangzheng Litter	Primary vs Secondary	1	0.298	2.465	0.236	0.023	**0.023**
Fangzheng Soil	Primary vs Secondary	1	0.840	3.132	0.281	0.007	**0.007**
Huangnihe Litter	Primary vs Secondary	1	0.566	2.640	0.248	0.007	**0.007**
Huangnihe Soil	Primary vs Secondary	1	1.140	7.952	0.498	0.007	**0.007**

Note: Bold values indicate significant statistical differences. Primary: Primary forest; Secondary: Secondary forest.

Table S4 Collembola species recorded in primary and secondary forests.

Family	Species	Forest
Entomobryidae	*Desoria* sp. 2	Primary and secondary forests
Isotomidae	*Folsomia octoculata*	Primary and secondary forests
Onychiuridae	*Heteraphorura seolagensis*	Primary and secondary forests
Hypogastruridae	*Ceratophysella* sp. 1	Primary and secondary forests
Hypogastruridae	*Ceratophysella* sp. 2	Primary and secondary forests
Odontellidae	*Superodontella* sp. 1	Primary and secondary forests
Neanuridae	*Friesea* sp. 1	Primary and secondary forests
Entomobryidae	*Homidia* sp. 5	Primary and secondary forests
Entomobryidae	*Sinella* cf. *umesaoi*	Primary and secondary forests
Tomoceridae	*Tomocerina* sp. 1	Primary and secondary forests
Onychiuridae	*Bionychiurus changbaiensis*	Primary and secondary forests
Odontellidae	*Superodontella* sp. 3	Primary and secondary forests
Isotomidae	*Folsomides* sp. 1	Primary and secondary forests
Odontellidae	*Superodntella* sp. 1	Primary and secondary forests
Isotomidae	*Folsomia* sp. 5	Primary and secondary forests
Entomobryidae	*Entomobrya* cf. *pulcherrima*	Primary and secondary forests
Neanuridae	*Deutonura* cf. *muscorum*	Primary and secondary forests
Entomobryidae	*Sinella* sp. 3	Primary and secondary forests
Entomobryidae	*Lepidocyrtus* sp. 2	Primary and secondary forests
Neanuridae	*Neanuridae* sp. 1	Primary and secondary forests
Entomobryidae	*Desoria* sp. 4	Primary and secondary forests
Neanuridae	*Koreanurina alba*	Primary and secondary forests
Tomoceridae	*Pogonognathellus* cf. *heterochrous*	Primary and secondary forests
Entomobryidae	*Desoria* sp. 13	Primary and secondary forests
Entomobryidae	*Willowsia* cf. *platani*	Primary and secondary forests
Tomoceridae	*Tomocerus* sp. 1	Primary and secondary forests
Entomobryidae	*Homidia* sp. 2	Primary and secondary forests
Tomoceridae	*Tomocerina* sp. 2	Primary forest
Neanuridae	*Coreanura* sp. 2	Primary forest
Dicyrtomidae	*Dicyrtoma* sp. 3	Primary forest
Neanuridae	*Deutonura* sp. 2	Primary forest
Neanuridae	*Lobellini* sp. 1	Primary forest
Odontellidae	*Superodontella* sp. 4	Primary forest
Dicyrtomidae	*Dicyrtoma* sp. 2	Primary forest
Onychiuridae	*Oligaphorura koreana*	Primary forest
Entomobryidae	*Homidia* sp. 3	Primary forest
Isotomidae	*Parisotoma* cf. *ekmani*	Primary forest
Isotomidae	*Proisotoma* sp. 1	Primary forest
Entomobryidae	*Desoria* sp. 12	Primary forest
Odontellidae	*Superodontella* sp. 5	Primary forest
Entomobryidae	*Desoria* sp. 7	Primary forest
Entomobryidae	*Homidia* sp.	Primary forest
Isotomidae	*Folsomia stella*	Primary forest
Entomobryidae	*Sinella* cf. *curviseta*	Primary forest
Entomobryidae	*Desoria* sp. 15	Primary forest
Neanuridae	*Deutonura* sp. 4	Primary forest

Entomobryidae	*Sinella* sp. 2	Primary forest
Tomoceridae	*Tomocerus laxalamella*	Primary forest
Isotomidae	*Subisotoma* sp. 2	Primary forest
Entomobryidae	*Desoria* cf. *hissarica*	Primary forest
Isotomidae	*Tetracanthella* cf. *wui*	Primary forest
Entomobryidae	*Desoria* sp. 11	Primary forest
Tomoceridae	*Tomocerus* cf. *nigrus*	Primary forest
Isotomidae	*Folsomia* cf. *stella*	Primary forest
Arrhopalitidae	*Arrhopalites* sp. 12	Primary forest
Isotomidae	*Folsomia ozeana* sp. 1	Primary forest
Entomobryidae	*Desoria choi*	Secondary forest
Neanuridae	*Deutonura* sp. 1	Secondary forest
Isotomidae	*Parisotoma* sp. 3	Secondary forest
Entomobryidae	*Sinella* sp. 4	Secondary forest
Neanuridae	*Coreanura* sp. 1	Secondary forest
Isotomidae	*Folsomides* sp.	Secondary forest
Isotomidae	*Folsomia inoculata* sp. 1	Secondary forest
Neanuridae	*Neanuridae* sp. 2	Secondary forest
Isotomidae	*Semicerura* sp.	Secondary forest
Entomobryidae	*Desoria* sp. 8	Secondary forest
Entomobryidae	*Desoria* sp. 6	Secondary forest
Entomobryidae	*Entomobrya* sp. 1	Secondary forest
Entomobryidae	*Homidia* sp. 11	Secondary forest
